# A Text-Book of Materia Medica, Therapeutics and Pharmacology

**Published:** 1900-09

**Authors:** 


A TEXT-BOOK OF MATERIA MEDICA, THERAPEUTICS AND PHARMACOLOGY.—By Geo. Frank Butler, Ph. G., M. D., Professor of Materia Medica and Clinical Medicine in the College of Physicians and Surgeons, Medical Department of the University of Illinois; Professor of General Medicine and Diseases of the Digestive System, Chicago Clinical School, etc. Third edition. Thoroughly revised. Pp. 874. Price, $4.00 and $5.00. Philadelphia: W. B. Saunders, 925 Walnut Street. 1900.

This work having passed to the third edition within the space of three years gives positive proof that the author's labors have not been in vain, that the book has merit and is appreciated to the fullest extent by the profession. Coming from a man ripe in years and experience; a man whose life-work has been in line with this subject, nothing else could be expected. Important changes have been made in this edition to bring it up to date. It is on the catalogues of many of the best medical schools of this country, which evidences its popularity. T. J. B.

